# Apolipoprotein E Genotype and the Relationship Between Chitinase 3–Like Protein 1 and Postoperative Delirium

**DOI:** 10.1093/geroni/igaa057.803

**Published:** 2020-12-16

**Authors:** Sarinnapha Vasunilashorn, Long Ngo, Sharon Inouye, Tamara Fong, Richard Jones, Simon Dillon, Towia Libermann, Edward Marcantonio

**Affiliations:** 1 Beth Israel Deaconess Medical Center, Boston, Massachusetts, United States; 2 Harvard University, Boston, Massachusetts, United States; 3 Institute for Aging Research / Hebrew SeniorLife, Boston, Massachusetts, United States; 4 Brown University, Providence, Rhode Island, United States

## Abstract

Apolipoprotein E (APOE) ɛ4 does not confer increased risk of delirium in older surgical patients; however, ɛ4 status modifies the relationship of C-reactive protein (CRP) with delirium: increased risk for delirium in ɛ4 carriers with high CRP. We examine whether APOE genotype modifies the established association between inflammatory marker chitinase-3-like protein-1 (CHI3LI/YKL-40) and delirium in patients without dementia age≥70 undergoing major non-cardiac surgery. We performed APOE genotyping using PCR, considering APOE ɛ4 vs. non-ɛ4 carriers. Plasma YKL-40, measured on postoperative day 2 by ELISA, was examined using sample-based quartiles (Q1-Q4). Delirium status was determined with daily interviews rating the Confusion Assessment Method, augmented by a validated chart review. We used generalized linear models adjusted for age, sex, surgery type, and stratified by APOE ɛ4 status. Among the 557 patients, 19% were APOE ɛ4 carriers, and 24% developed postoperative delirium. The YKL-40-delirium relationship differed by APOE status. Among APOE non-ɛ4 carriers, we found a significant relationship between YKL-40 and delirium (relative risk [RR](95% confidence interval [CI] for YKL-40 Q4 vs. Q1: 2.6(1.4-4.9) and Q3 vs. Q1: 2.3(1.2-4.5); p-trend<.01). Among APOE ɛ4 carriers, YKL-40 was not significantly associated with delirium (RR(95% CI) for YKL-40 Q4 vs. Q1: 2.0(0.6-6.6) and Q3 vs. Q1:1.1(0.3-3.5); p-trend=0.37). APOE non-ɛ4 carriers may have increased risk of delirium conferred by post-surgical inflammation specific to the type 2 immune response (high YKL-40). These results differ from prior results with CRP, and raise the possibility that APOE genotype may interact at different points in the inflammatory pathway leading to delirium.

